# The constancy of gene conservation across divergent bacterial orders

**DOI:** 10.1186/1756-0500-2-2

**Published:** 2009-01-07

**Authors:** Olin K Silander, Martin Ackermann

**Affiliations:** 1 Institute of Integrative Biology, ETH Zurich, Zurich, Switzerland 8092

## Abstract

**Background:**

Orthologous genes are frequently presumed to perform similar functions. However, outside of model organisms, this is rarely tested. One means of inferring changes in function is if there are changes in the level of gene conservation and selective constraint. Here we compare levels of gene conservation across three bacterial groups to test for changes in gene functionality.

**Findings:**

The level of gene conservation for different orthologous genes is highly correlated across clades, even for highly divergent groups of bacteria. These correlations do not arise from broad differences in gene functionality (e.g. informational genes vs. metabolic genes), but instead seem to result from very specific differences in gene function. Furthermore, these functional differences appear to be maintained over very long periods of time.

**Conclusion:**

These results suggest that even over broad time scales, most bacterial genes are under a nearly constant level of purifying selection, and that bacterial evolution is thus dominated by selective and functional stasis.

## Background

We are interested in whether the functional importance of orthologous genes changes across bacterial taxa. We pose a simple question: if a gene plays an important role for the functioning of a bacterium, is the orthologue of that gene in a distantly related bacterium also particularly important? To measure functional importance, we look at how strongly genes are conserved over time. If a group of orthologous genes are well conserved across different taxa, this implies that strong purifying selection is acting to maintain these genes. Similarly, if a group of orthologous genes are lost quickly across different taxa, this implies that purifying selection is acting only weakly to maintain these genes. If the strength of purifying selection for individual genes does not change across bacterial groups (i.e. if there is a correlation in the level of orthologue conservation), this implies that the functional importance of most orthologous genes does not change quickly. On the other hand, if there is little correlation in the level of gene conservation across bacterial groups, this implies that the functional importance of many orthologues changes often, perhaps because of differences in the genetic backgrounds of organisms (e.g. compensating mechanisms at other loci).

Here we show that between three bacterial clades, the levels of conservation for specific orthologues are highly correlated. This correlation remains even when examining subgroups of functionally similar genes, such as genes involved in ribosome function. This suggests that despite large differences in genetic background, the strength of selection acting to maintain any specific orthologue remains approximately constant, and that most genes maintain their specific functionality over long periods of time.

We used stochastic character mapping [1] to calculate a measure of gene conservation that accounts for phylogenetic relatedness between taxa. Briefly, for each protein coding gene in *E. coli K12 W3110*, we determined whether an orthologous gene was present or absent for all other bacteria with fully sequenced genomes (447 other genomes in total). Together with information on the phylogenetic history, these data were used to calculate a parameter for each orthologue that reflects the rate (probability per unit time) that the orthologue the orthologue will be lost or gained along a branch (see Additional file 1). Because this parameter value is mostly determined by how quickly an orthologue is lost over time, we term this parameter the rate of orthologue loss (ROL). Low ROL values imply that along a branch, there is a low probability of that orthologue being lost. High ROL values imply that along a branch, there is a high probability that the orthologue will be lost. For each orthologue, one ROL was calculated for all branches in a clade.

## Methods

All genomes were downloaded from the NCBI database in May of 2007 (Additional file 2), and a phylogeny was constructed using a concatenated set of 73 conserved orthologues (Additional files 3 and 4). The program SIMMAP [2] was used to calculate all ROL values (Additional file 5), and gene functional classes were divided using MultiFun [3]. For detailed materials and methods, see Additional file 1.

## Results

### Constancy of ROL across different bacterial clades

**Figure 1:**
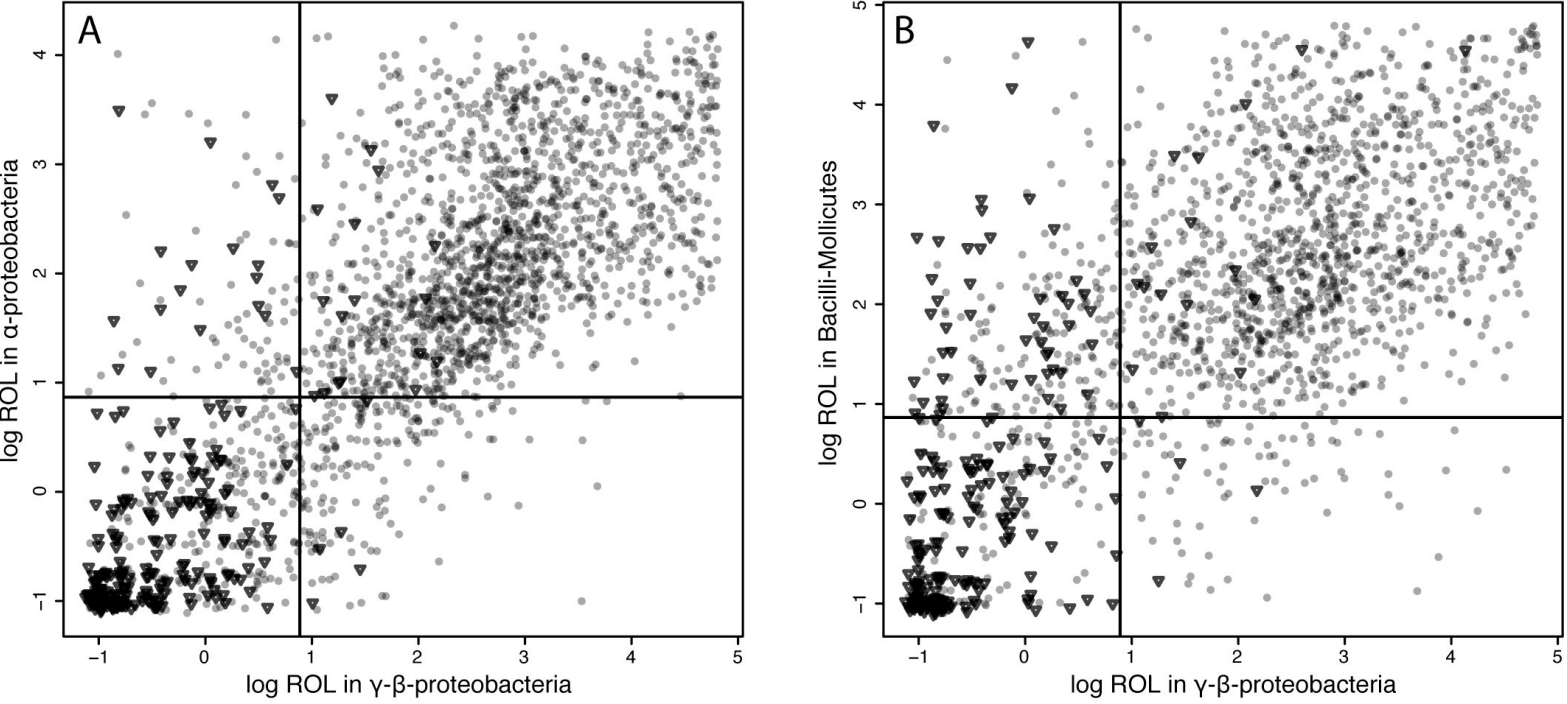
**Relationship between gene ROLs for different bacterial clades**. A) γ-β-proteobacteria versus α-proteobacteria. Each point indicates the estimated ROL for that gene (orthologue set) in γ-β-proteobacteria as opposed to α-proteobacteria. Essential genes are shown as inverted triangles; nonessential genes as grey circles. There is a highly significant relationship between individual ROL in the two clades (r = 0.820, p < 0.0001). This remains true even when essential genes are removed from consideration (r = 0.734, p < 0.0001). The lines indicate the cut-off ROL values used in Table 1. B) γ-β-proteobacteria versus Bacilli and Molllicutes. Each point indicates the estimated ROL for that set of orthologues in γ-β-proteobacteria as opposed to Bacilli and Molllicutes. Essential genes in *E. coli *are shown as inverted triangles; nonessential genes as grey circles. There is a highly significant relationship between ROLs for individual orthologues in the two clades (r = 0.700, p < 0.0001). This remains true even when orthologues that are essential in *E. coli *are removed from consideration (r = 0.576, p < 0.0001). The lines indicate the cut-off ROL values used in Table 1.

We tested whether the ROL values for specific orthologues change between different clades of bacteria. We calculated the ROL values for orthologous genes in three bacterial groups: the γ – and β-proteobacteria; the α-proteobacteria, which are the sister clade of the γ-β-proteobacteria and diverged approximately 2.5 billion years ago [4]; and the Bacilli-Molllicutes clade, which diverged from the γ-β-proteobacteria just over three billion years ago [4]. For all of these clades, we found that the ROL values for orthologous genes were highly correlated (Fig. 1; r^2 ^= 0.673, Pearson's ρ = 0.756 for γ-β-proteobacteria versus α-proteobacteria; r^2 ^= 0.488, Pearson's ρ = 0.628 for γ-β-proteobacteria versus Bacilli-Molllicutes; all data are listed in Additional file 5).

A simple explanation for the high correlation between ROL values is that it is driven by differences in gene essentiality: essential genes will be strongly conserved, while nonessential genes will be weakly conserved. To test this, we divided the genes into essential and nonessential groups, based on the experimental results from two recent studies that used either *E. coli K12 MG1655 *[5] or *E. coli K12 BW25113 *[6]. We disregarded any discrepancies in essentiality annotation between the two studies and focused only on those genes for which they agree on the classification of essentiality. We found that even when excluding orthologues that are classified as essential in *E. coli*, the correlations remained very high (Fig. 1; r^2 ^= 0.539 and r^2 ^= 0.332, respectively).

**Figure 2:**
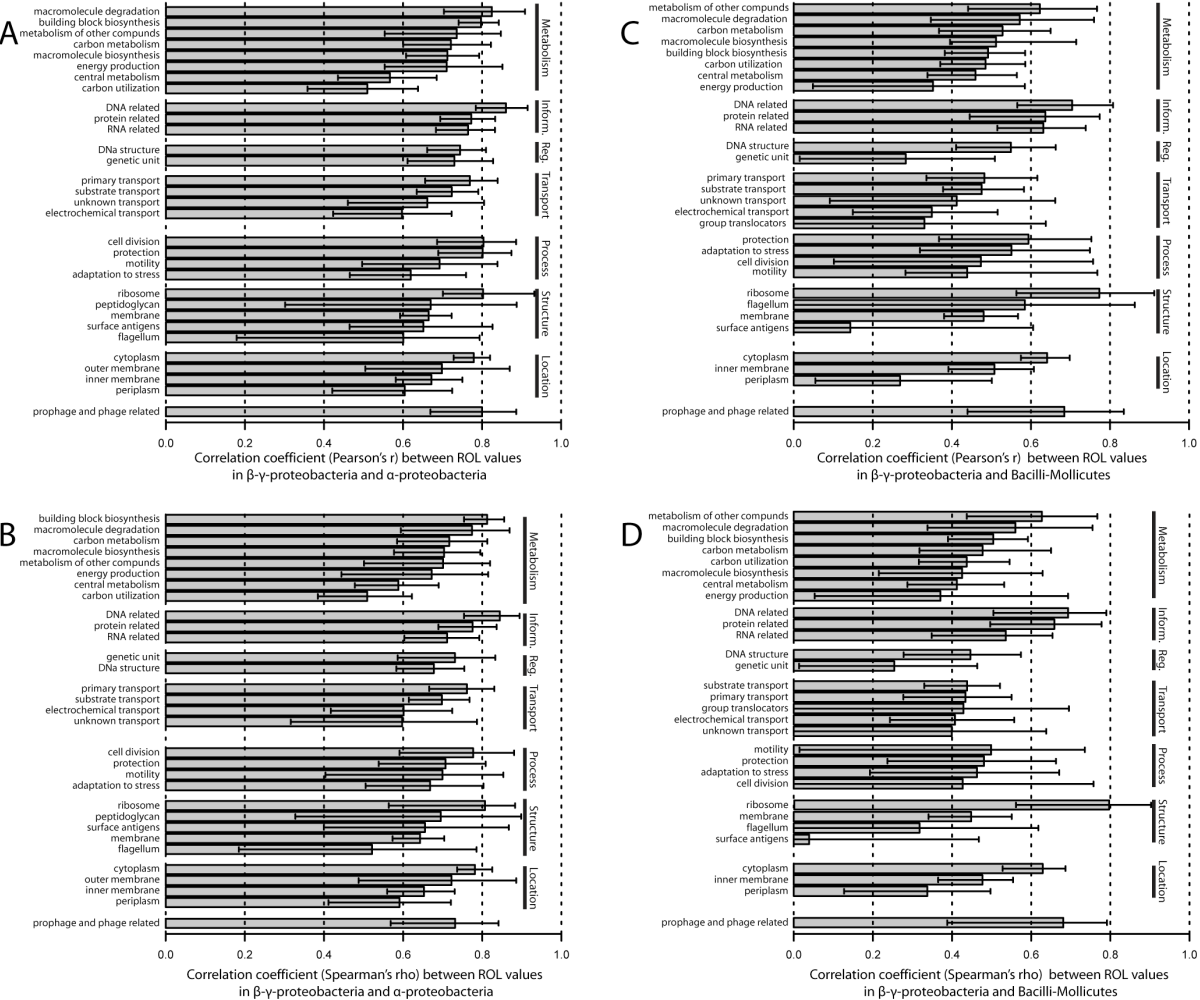
**The correlations between ROL values is not driven by broad differences among different functional classes of genes or differences between essential and nonessential genes**. For each functional class (as defined by MultiFun [3]) the parametric and nonparametric correlation coefficient between the ROL for nonessential orthologous genes in the γ-β-proteobacteria, α-proteobacteria, and Bacilli-Mollicutes was calculated. Only MultiFun classes for which ROL values existed for at least 20 genes are shown. Several functional classes are abbreviated, as follows: Inform., Information transfer (Class 2); Reg., Regulation (Class 3); Process, Cell processes (Class 5); Structure, Cell structure (Class 6); Location, Location of gene products (Class 7); genetic unit, genetic unit regulated (3.3); primary transport, primary active transporters (4.3); electrochemical transport, electrochemical potential driven transport (4.2); prophage and phage related, prophage genes and phage related functions (8.1). Error bars were calculated by bootstrapping pairs of ROL values within each class 1000 times [15]. A) Parametric correlation coefficients between γ-β-proteobacteria and α-proteobacteria ROL values for each functional class. B) Nonparametric correlation coefficients (Spearman's ρ) between γ-β-proteobacteria and α-proteobacteria ROL values for each functional class. C) Parametric correlation coefficients between γ-β-proteobacteria and Bacilli-Mollicutes ROL values for each functional class. D) Nonparametric correlation coefficients (Spearman's ρ) between γ-β-proteobacteria and Bacilli-Mollicutes ROL values for each functional class.

A second explanation for the high correlation between ROL values is that it is driven by differences between functional classes of genes. For example, informational orthologues may be highly conserved, whereas genes involved in metabolic functions may be less conserved. To test this hypothesis, we calculated the correlation coefficients for ROL values of orthologues within single functional classes of genes as delineated by MultiFun [3] (see Additional file 1). We found that within MultiFun classes, ROL values between bacterial groups were again highly correlated, even when considering only nonessential genes. The r^2 ^values between γ-β-proteobacteria and α-proteobacteria varied from 0.740 (for information transfer genes related to DNA, MultiFun class 2.1) to 0.260 (for carbon utilization genes, class 1.1) (Fig. 2). The r^2 ^values between γ-β-proteobacteria and Bacilli-Mollicutes varied from 0.600 (for structural genes in the ribosome, MultiFun class 6.6) to 0.020 (for structural genes responsible for surface antigens, class 6.3) (Fig. 2). Together, these data suggest that ROL values remain constant over long stretches of time, on the order of billions of years, and that this constancy is driven neither by broad differences in gene functionality, nor differences in gene essentiality, but by specific differences in gene function.

### The relationship between ROL and gene essentiality

**Figure 3:**
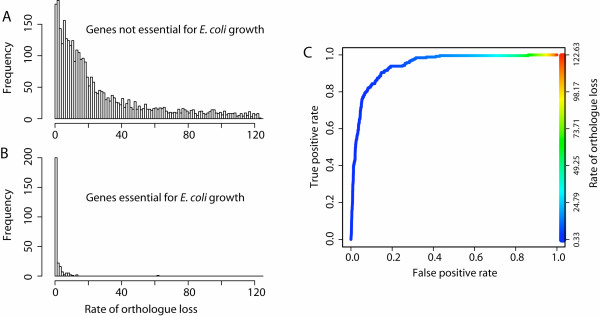
**Rates of orthologue loss (ROL) for *E. coli *genes annotated as essential are lower than for those annotated as nonessential**. ROL is a rate parameter that reflects both the numbers of gene losses and gene gains (see Materials and Methods). A) Histograms of ROL values for nonessential genes in *E. coli*. B) Histograms of ROL values for essential genes in *E. coli*. On average, essential genes have a drastically lower ROL than nonessential genes. C) Quantification of the relationship between ROL and essentiality. The receiver operator characteristic (ROC) curve describes the relationship between the fraction of false positives and the fraction of true positives when using ROL as the discriminating variable. A measure of how well ROL predicts essentiality is the area under this curve (the AUC). In this case, the AUC is 0.946; if ROL were perfectly predictive of essentiality, the AUC would be 1.0.

We have assumed above that the level of gene conservation reflects the strength of purifying selection acting on a gene: well-conserved genes are under strong purifying selection, while less conserved genes experience only weak purifying selection. Here we test this assumption by asking how well our measure of gene conservation, ROL, corresponds to growth phenotypes, which we know to be under selection. Specifically, if the deletion of a gene causes lethality even under benign laboratory conditions, then the loss of this gene is almost certainly lethal in the natural environment and is thus under strong purifying selection. We first ask, then, how well ROL values correlate with annotations of gene essentiality. The ROL values for essential and nonessential genes are shown in Fig. 3A. On average, genes that have been classified as essential in *E. coli K12 *have a dramatically lower ROL than non-essential genes.

To quantify the relationship between ROL and essentiality, we used a receiver operator characteristic (ROC) curve. This curve describes the relationship between the fraction of false positives and the fraction of true positives when using ROL to discriminate between essential and nonessential genes. One means of quantifying this relationship is by calculating the area under the ROC curve (the AUC), which is equivalent to the probability that a randomly chosen essential gene will have a lower ROL than a randomly chosen nonessential gene [7]. If ROL were perfectly predictive of gene essentiality, the AUC would be 1.0; the AUC for this analysis was 0.947 (Fig. 3B), and strongly suggests that ROL values do reflect the strength of purifying selection acting on a gene.

We also asked whether ROL values and the quantitative effects of gene deletions are correlated. Using data on growth yield in rich media of deletion mutants [6], we found a small but highly significant relationship between a gene's ROL value and the growth yield of that deletion strain (Fig. S2; r^2 ^= 0.0628, p < 0.0001; Spearman's ρ = 0.127, p < 0.0001). Again, this suggests that ROL values reflect the strength of purifying selection acting on a gene.

## Conclusion

**Figure 4:**
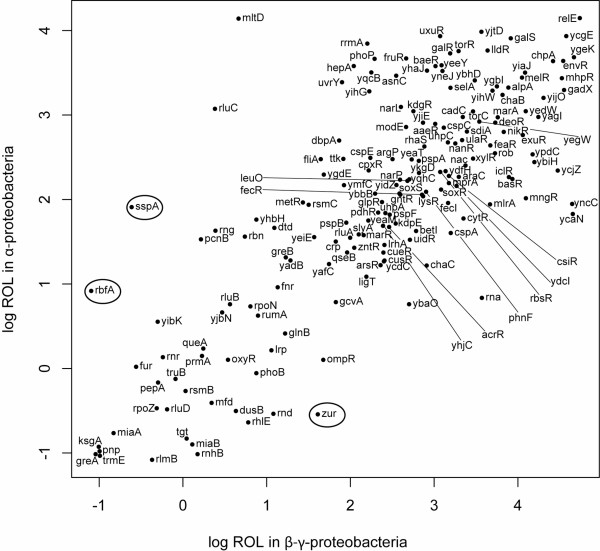
**Correlation between gene ROL values for information transfer RNA related ((MultiFun class 2.2) nonessential genes**. The ROL values for γ-β-proteobacteria and α-proteobacteria are plotted. Each point indicates the estimated ROL value for that gene (orthologue set) in γ-β-proteobacteria as opposed to α-proteobacteria; the *E. coli *gene name is plotted next to the point. There is a highly significant relationship between individual ROL in the two clades (r^2 ^= 0.584, p < 0.0001; Spearman's ρ = 0.71, p < 0.0001). Three genes that fall far from the expected values are circled; the phylogenetic pattern of conservation for these genes is shown in Figure 5.

**Figure 5:**
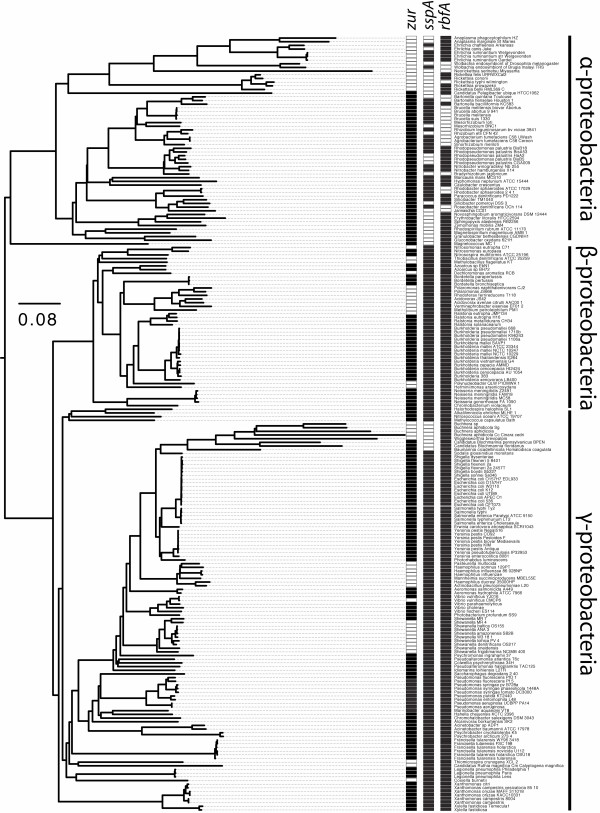
**Phylogenetic pattern of conservation for *zur*, *sspA*, and *rbfA***. Unlike most RNA related genes (Fig. 5), these three genes have different patterns of conservation, and thus different ROL values, when compared between γ-β-proteobacteria and α-proteobacteria. The figure shows the phylogeny of the α-γ-β-proteobacteria together with *Magnetococcus *MC1, with the names of each taxa listed; this is a subset of the taxa presented in Figure S1. The columns show the conservation patterns for orthologues of the *E. coli *RNA related genes involved in information transfer (*zur*, *sspA*, and *rbfA*, respectively). Black boxes indicate orthologue presence, while white indicate absence. Orthologues of *zur*, a transcriptional regulator for zinc uptake, are almost completely conserved in α-proteobacteria, and were likely lost only a single time in the intracellular *Rickettsia *lineage. This can be contrasted with the pattern of conservation in the γ-β-proteobacteria, from which *zur *orthologues have been lost multiple times, and on relatively quick timescales: an orthologue appears in *Shewanella sp*. MR-7, but is absent from *Shewanella sp*. MR-4. Orthologues of *sspA*, associated with the RNA ROLymerase, are conserved almost completely in the γ-β-proteobacteria, having been lost only from the intracellular *Candidatus-Buchnera *lineage. However, in the α-proteobacteria *sspA *orthologues have been lost multiple times, and over short periods of time in some cases: an orthologue appears to be present in *Mesorhizobium loti*, but absent in *Mesorhizobium *BNC1. Finally, orthologues of *rbfA*, associated with the ribosome, are perfectly conserved in the γ-β-proteobacteria, but have been lost multiple times from the α-proteobacteria. The first case (*zur*) is consistent with an increased level of constraint within the α-proteobacteria, as this gene also has a high ROL value in the Bacilli-Mollicutes; in the case of *rbfA*, a relaxation in selective constraint within the α-proteobacteria seems likely, as orthologues of this gene are also highly conserved in the Bacilli-Mollicutes clade (*sspA *has no orthologues in the Bacilli-Mollicutes).

We have shown that ROL values for specific orthologues are correlated over long broad evolutionary distances, and that these correlations remain strong even within specific functional classes of genes and for genes that are not essential for cellular viability. In other words, the constancy of the level gene conservation across bacterial orders seems to result from specific differences in gene function. The strength of the correlations we find here are of similar magnitude to one found in a previous study of correlations between protein evolutionary rates within the Chlamydiaceae [8]. Notably, the Chlamydiaceae are far more closely related than the clades considered here, so a high correlation should not be surprising. However, we have also considered selection on a more general scale (gene presence versus gene absence), which likely increases the strength of the correlations. Interestingly, for some orthologues, ROL values have changed considerably across taxonomic groups (we show three examples in Figs. 4 and 5). We propose that these genes have changed in functional importance, resulting in either increased or decreased purifying selection.

Some essential *E. coli *genes have orthologues that are consistently lost at high rates among other γ-β-proteobacteria, α-proteobacteria, and Bacilli-Mollicutes, contrary to the high level of conservations expected for essential genes. This is not due to these genes only being essential in *E. coli *and nonessential in other taxa. In Table 1 we show a list of genes that are essential in *E coli K12 *and which have high ROL values (greater than 2.4 in all three bacterial groups studied (Fig. 1)), together with data from an empirical study of gene essentiality in the γ-proteobacterium *Acinetobacter baylyi *[9]. Of nine genes with an orthologue in *Acinetobacter*, eight are also essential in *Acinetobacter*. This suggests, surprisingly, that some genes, despite being essential, are lost frequently, and is consistent with the view that compensation at other sites in the genome may occur even for "essential" functions.

**Table 1:** Essential *E. coli *genes with high ROL values are also essential in *Acinetobacter*.

K12 gene	Gene function	ROL γ-β	ROL α	ROL B-M	Essential
asd	ASA dehydrogenase	3.07	n.a.	n.a.	DB
can	carbonic anhydrase	4.07	11.67	n.a.	-
degS	serine endoprotease	8.63	9.54	7.77	NON
dnaC	DNA biosynthesis protein	62.30	n.a.	94.06	-
fabA	HD thioester dehydrase	2.78	2.42	n.a.	-
fabB	3-oxoacyl-[acp] synthase	3.55	2.75	n.a.	DB
fbaA	FBP aldolase	3.21	n.a.	n.a.	-
fldA	flavodoxin 1	4.73	22.92	16.86	-
ftsE	transporter subunit	3.05	2.48	8.79	-
ftsK	chromosome partitioning	7.20	2.55	10.41	ESS
ftsL	cell division protein	4.59	n.a.	n.a.	-
ftsN	cell division protein	12.64	n.a.	n.a.	-
ftsX	transporter subunit	2.87	13.32	9.10	-
hemD	uroporphyrinogen synthase	2.75	n.a.	n.a.	DB
hemG	protoporphyrin oxidase	8.47	n.a.	n.a.	-
holB	DNA ROL III subunit	5.08	19.02	32.24	-
lolB	chaperone for lipoproteins	2.67	n.a.	n.a.	ESS
lolE	lipoprotein transporter	7.92	5.89	55.12	-
mreD	cell wall structural complex	3.53	n.a.	n.a.	-
mukB	chromosome partitioning	13.42	n.a.	94.78	-
mukE	chromosome partitioning	7.41	n.a.	n.a.	-
mukF	chromosome partitioning	7.16	n.a.	n.a.	-
nrdA	RDP reductase subunit	4.91	n.a.	n.a.	-
nrdB	RDP reductase subunit	3.03	5.75	n.a.	ESS
plsB	O-acyltransferase	5.09	n.a.	n.a.	ESS
plsC	acyltransferase	4.06	5.79	32.81	-
psd	decarboxylase	3.28	36.74	13.10	ESS
pssA	phosphatidylserine synthase	7.12	n.a.	n.a.	-
rlpB	minor lipoprotein	8.13	n.a.	n.a.	-
secM	regulator of secA	10.80	n.a.	n.a.	-
yejM	hydrolase, inner membrane	6.76	n.a.	n.a.	-
yrbK	conserved protein	8.94	n.a.	n.a.	-
yrfF	inner membrane protein	7.93	n.a.	n.a.	-
zipA	cell division protein	3.33	n.a.	n.a.	-

Only those *E. coli K12 *genes with high ROL values in γ-β-proteobacteria, α-proteobacteria, and Bacilli-Mollicutes (ROL values greater than 2.4 in all three groups (**Fig. 1**)) are shown here. Of these 34 genes, nine have an orthologue in *Acinetobacter*, and eight of these have an essential function in *Acinetobacter*. Many of the genes lost at high rates from γ-β-proteobacteria have orthologues in fewer than 10% of the α-proteobacteria or Bacilli-Mollicutes; these are indicated by n.a. ESS, genes for which no deletion mutants were isolated in *Acinetobacter*; DB, genes in which only "double band" mutants containing chromosomal duplications were isolated in *Acinetobacter *(and which are thus likely to be essential); NON, genes for which deletion mutants were isolated in *Acinetobacter*, and which are thus considered to be non-essential; [9]. '-', genes without an orthologue in *Acinetobacter*.

Many of the essential genes that are lost at high rates are recent innovations. Considering those genes that are essential in *E. coli K12 *but are lost at high rates from other γ-β-proteobacteria, 44% (18 out of 41) have a distribution restricted to the γ-β-proteobacteria and are thus likely to be relatively recent additions to the genomic repertoire. In contrast, of the essential genes with low ROL values (less than 2.4), only 0.9% (2 out of 222) are restricted to the γ-β-proteobacteria. Previous work has shown that recently acquired genes tend to be incorporated at the edge of the cellular network [10]. Such peripheral genes may thus be more easily removed from the genome, with fewer interactions to compensate.

These results confirm and extend previous studies that have investigated the relationship between essentiality and gene conservation [11-13]. However, here we have used a phylogenetically corrected measure of gene conservation (ROL). Additionally, we have found that the ability of orthologue conservation to predict gene essentiality is far higher than has previously been realized [11], most likely due to the lower accuracy of earlier datasets. Finally, we have shown for the first time a correlation between gene conservation and quantitative measures of deletion phenotypes (growth yield, Fig. S2).

Our metric of gene conservation, which takes into account phylogenetic history, provides a considerable improvement over simpler measures such as the fraction of taxa that retain a specific orthologue (retention). Using retention to predict essentiality yields an AUC of 0.937, meaning that essential genes are incorrectly ranked higher than nonessential genes 6.3% of the time. Using ROL, the misclassified fraction is reduced to 5.3%, a reduction of 16% in the error rate. ROL has the additional advantage of being based on a specific evolutionary model, which itself may provide biological insights, for example into the relative rates of gene loss versus horizontal transfer (i.e. the ratio of gene loss versus gene gain in lineages).

Finally, we note that high-throughput experimental assessments of gene essentiality are prone to both false positive and false negative results (i.e. annotating a non-essential gene as essential and vice versa). The level of agreement on essentiality between the two most recent studies of gene essentiality [5,6] is similar to the level of agreement between both studies and ROL (all are between 94% and 95%), and far greater than between the first experimental study of gene essentiality [14] and the latter two experimental studies. This suggests that ROL may be a valid and useful means of cross-validating experimental studies in order to find genes likely to be false positives or false negatives, which could then be reexamined.

## Abbreviations

ROC: receiver operator characteristic; AUC: area under the ROC curve; ROL: rate of orthologue loss; *W3110*: *E. coli K12 W3110*.

## Competing interests

The authors declare that they have no competing interests.

## Authors' contributions

OKS and MA conceived of the study. OKS performed the bioinformatic and phylogenetic analyses and drafted the manuscript. MA edited the manuscript.

## Supplementary Material

Additional file 1 **Detailed materials and methods**. Detailed materials and methods are outlined here, including the methods used to build the phylogeny and the method used to infer ROL values. Three supplementary figures are also included, showing the phylogeny, the relationship between ROL and deletion strain growth phenotype, and the sensitivity of ROL values to changes in the ratio of gene loss to gene gain. Click here for file

Additional file 2 **Bacterial and Archaeal genomes used to construct orthologue sets**. All taxa used in the phylogenetic analysis are listed here; the taxon names are shown as written in the NCBI database. Click here for file

Additional file 3 **Universal orthologues used to construct the distance based phylogeny**. The list of universally distributed genes used to construct the phylogeny are listed here; the top row indicates the *E. coli *orf. Below the *E. coli *orf, the orthologous reading frame in each genome is listed. Click here for file

Additional file 4 **Full amino acid alignment used to construct phylogeny**. This Phylip format file shows the full amino acid alignment used to construct the distance based phylogeny. The taxa names are abbreviated; the full names of each taxon are listed in Additional file 2. Click here for file

Additional file 5 **Essentiality annotations and ROL values for all *E. coli *genes**. The data here show the annotations of essentiality and non-essentiality, as well as the ROL values calculated for each orf in *E. coli*. The data are listed in tab-delimited columns, as follows: *E. coli *orf name; Blattner number used by PEC, Blattner number used by Keio study; whether the gene is annotated as essential by the PEC study (1 = essential, 2 = nonessential, 4 = unknown); whether the gene is annotated as essential by the Keio study; the ROL value calculated when using all 448 bacterial taxa, the ROL when using only γ-β-proteobacteria (NA: orthologues were present in fewer than 10% of the taxa); the ROL when using only α-proteobacteria; the ROL when using only Bacilli-Mollicutes. Click here for file

## References

[B1] Huelsenbeck JP, Nielsen R, Bollback JP (2003). Stochastic mapping of morphological characters. Systematic Biology.

[B2] Bollback JP (2006). SIMMAP: Stochastic character mapping of discrete traits on phylogenies. Bmc Bioinformatics.

[B3] Serres MH, Riley M (2000). MultiFun, a multifunctional classification scheme for Escherichia coli K-12 gene products. Microbial & comparative genomics.

[B4] Battistuzzi FU, Feijao A, Hedges SB (2004). A genomic timescale of prokaryote evolution: insights into the origin of methanogenesis, phototrophy, and the colonization of land. Bmc Evolutionary Biology.

[B5] Kato JI, Hashimoto M (2007). Construction of consecutive deletions of the Escherichia coli chromosome. Molecular Systems Biology.

[B6] Baba T, Ara T, Hasegawa M, Takai Y, Okumura Y, Baba M, Datsenko KA, Tomita M, Wanner BL, Mori H (2006). Construction of Escherichia coli K-12 in-frame, single-gene knockout mutants: the Keio collection. Molecular Systems Biology.

[B7] Fawcett T (2006). An introduction to ROC analysis. Pattern Recogn Lett.

[B8] Jordan IK, Kondrashov FA, Rogozin IB, Tatusov RL, Wolf YI, Koonin EV (2001). Constant relative rate of protein evolution and detection of functional diversification among bacterial, archaeal and eukaryotic proteins. Genome Biology.

[B9] de Berardinis V, Vallenet D, Castelli V, Besnard M, Pinet A, Cruaud C, Samair S, Lechaplais C, Gyapay G, Richez C, Durot M, Kreimeyer A, Le Fèvre F, Schächter V, Pezo V, Döring V, Scarpelli C, Médigue C, Cohen GN, Marlière P, Salanoubat M, Weissenbach J (2008). A complete collection of single-gene deletion mutants of Acinetobacter baylyi ADP1. Molecular Systems Biology.

[B10] Pal C, Papp B, Lercher MJ (2005). Adaptive evolution of bacterial metabolic networks by horizontal gene transfer. Nature Genetics.

[B11] Gustafson AM, Snitkin ES, Parker SCJ, DeLisi C, Kasif S (2006). Towards the identification of essential genes using targeted genome sequencing and comparative analysis. Bmc Genomics.

[B12] Jordan IK, Rogozin IB, Wolf YI, Koonin EV (2002). Essential genes are more evolutionarily conserved than are nonessential genes in bacteria. Genome Research.

[B13] Fang G, Rocha E, Danchin A (2005). How essential are nonessential genes?. Molecular Biology and Evolution.

[B14] Gerdes SY, Scholle MD, Campbell JW, Balazsi G, Ravasz E, Daugherty MD, Somera AL, Kyrpides NC, Anderson I, Gelfand MS, Bhattacharya A, Kapatral V, D'Souza M, Baev MV, Grechkin Y, Mseeh F, Fonstein MY, Overbeek R, Barabasi AL, Oltvai ZN, Osterman AL (2003). Experimental determination and system level analysis of essential genes in Escherichia coli MG1655. Journal of Bacteriology.

[B15] Efron B, Tibshirani RJ (1993). An introduction to the Bootstrap.

