# Supplementary material for: The constancy of gene conservation across divergent bacterial orders
Source: BMC Res Notes. 2009 Jan 7;2:2. doi: 10.1186/1756-0500-2-2 (PMC2633337; doi:10.1186/1756-0500-2-2)
Supplement: Additional file 1 — Detailed materials and methods. Detailed materials and methods are outlined here, including the methods used to build the phylogeny and the method used to infer ROL values. Three supplementary figures are also included, showing the phylogeny, the relationship between ROL and deletion strain growth phenotype, and the sensitivity of ROL values to changes in the ratio of gene loss to gene gain. [file 1756-0500-2-2-S1.pdf]

Detailed materials and methods

Orthologue definitions. The protein coding genes from 448 fully sequenced bacterial species (**Additional file 2**) were downloaded from the NCBI database in May of 2007. The *E. coli K12 W3110* genome (AP009048) was used to search all other genomes for orthologous genes, which were defined using a reciprocal shortest distance method [1] (the *W3110* genome is the closest sequenced genome to *E. coli K12 BW25113*, which used in the single gene deletion study [2]). Briefly, each *E. coli* protein-coding gene was blasted against all protein-coding genes in all other genomes. For each genome, the top ten hits having an e-value score less than 0.1 and which differed by less than 40% in length, were retained. Each of these was aligned individually to the *W3110* gene, and using PAML, the evolutionary distance to each was calculated. The gene having the shortest evolutionary distance was retained as a hypothetical orthologue, after which the reciprocal process was performed, in which the hypothetical orthologue was blasted against the *W3110* genome, aligned against the top ten hits, and for each, the evolutionary distance was calculated. If the original gene was found to be the most closely related gene, then these two genes were considered orthologues. No orthologue data were collected for any of the 74 protein coding genes in *W3110* that are annotated as IS elements.

Phylogeny construction. All sets of orthologous genes that were present in at least 99% (444) of the fully sequenced bacterial species were used to construct a phylogenetic tree (73 orthologue sets in total, listed in **Additional file 3**). Four archaeal species (*Archaeoglobus fulgidus*, *Methanococcus jannaschii*, *Nanoarchaeum equitans*, and

Sulfolobus solfataricus) were used to root the tree. The orthologue sets were individually aligned using MUSCLE v3.6 [3], and these alignments were concatenated and poorly aligned regions were cleaned up using Gblocks v0.91b [4] with the maximum number of contiguous non-conserved residues set to 8, the minimum length of a block set to 2, and intermediate gap positions allowed. The full alignment file is provided in **Additional file 4**.

Phylip v3.65 [5] was used to calculate a distance matrix from the full supermatrix of amino acid positions (18,666 positions), with a JTT model of amino acid substitution and gamma-distributed rate variation across sites. FastME2.0 [6], which employs a minimum evolution method, was used to build the phylogenetic tree. FigTree v1.0 [7] was used for tree visualization. The topology that we inferred for the phylogenetic relationships largely agrees with previous studies (**Fig. S1**), although we briefly note the following contrasts: we find a weak grouping of the Actinobacteria with Deinococcales, Chloroflexi, and Cyanobacteria, in agreement with one recent study [8] but in disagreement with a second [9]. We find that the earliest branching clades are the Aquifex and Thermotoga clades, again, more closely agreeing with Pisani et al. than Ciccarelli. We find that the Spirochaetes, Chlorobi, and Chlamydiae group together [8], with Planctomycetes also within this grouping. We find the ε-proteobacteria to be the most diverged proteobacterial group; additionally, we do not find the δ-proteobacteria to be monophyletic, as the Acidobacteria appear within the clade. Finally, we note that Magnetococcus does not group strongly with any proteobacterial group, instead falling as

a deeply rooted sister taxa to the α-proteobacteria, in agreement with the most recent study [10].

Rate of orthologue loss. We did not collect orthologue data for any *W3110* gene that was annotated as an insertion sequence, which currently lacks a Blattner number, or which was not annotated in the NCBI file downloaded in May 2007. The rate of orthologue loss for each set of orthologues was calculated using SIMMAP 1.0 (Beta 2.3.2) [11], which uses a method of stochastic character mapping first described by Huelsenbeck at al. [12]. Briefly, for each protein coding gene in *E. coli K12 W3110*, we defined orthologues as present or absent for all bacterial taxa with fully sequenced genomes (447 other genomes in total) using the reciprocal smallest distance algorithm outlined above. This data was then coded as a binary character matrix, with one indicating orthologue presence and zero indicating orthologue absence in each taxa. These data, together with information on the phylogenetic relationships between the bacterial taxa were used to calculate a rate of change for each character, which we term rate of orthologue loss (ROL). The ROL value for each set of orthologues is a rate parameter that reflects the rate at which a gene is lost and gained across a group of bacteria. However, for the range of parameters we considered, ROL values largely indicate how quickly orthologue losses occur across the phylogeny (see below). In each analysis, ROL values were calculated only for *E. coli* genes with orthologues present in greater than 10% of the taxa. For the γ-β-proteobacteria (153 total taxa), the total number of orthologue sets considered was 3670; for α-proteobacteria (59 total taxa), the total number of orthologue sets was 2328, and for

the Bacilli and Mollicutes clade (89 total taxa), the total number of orthologue sets was 1866.

For all SIMMAP analyses, a fixed prior on the bias in character transition rates from 1 (orthologue presence) to 0 (orthologue absence) was used; this bias favored gene loss over gene gain (i.e. gene loss over horizontal gene transfer) by a ratio of 9:1. Changing the bias parameter had very little effect on the relationship between ROL and gene essentiality (**Fig. S3**). The prior on ROL (the rate parameter) was a broad gamma distribution, with size parameter $\alpha = 1.25$, shape parameter $\beta = 0.05$, and the number of discrete rate categories $k = 100$. Again, the relationship between ROL and gene essentiality was affected very little by changes in the shape of the prior (data not shown). To calculate the ROL values for each gene, at least 100 realizations were performed.

The phylogenetic measure of gene conservation that we use here (ROL) is a rate parameter that reflects both gene loss and gene gain. We found that using only the numbers of gene losses or gene gains resulted in both less accurate and less robust predictions of gene essentiality in *E. coli* (**Fig. S3**). Since our intent was to choose a phylogenetic measure that most closely reflected the action of selection, we used the rate measure, which we refer to as the rate of orthologue loss (ROL).

Gene essentiality and quantitative effects of gene deletions. Measurements of gene essentiality were derived from two experimental studies in *E. coli*: a large-scale targeted gene deletion study (Keio) [2], and a long-range deletion study (Profiling the *E. coli*

Chromosome, PEC) [13]. The quantitative measures of the consequences of gene deletions were taken from the experimentally measured growth yields in rich media from [2].

When looking at overlaps between the classifications of essentiality, we considered all proteins annotated in either study, with the following exceptions. The ancestral K12 is missing the gene *rph* (b3643) so it was excluded from all analyses. Second, seven genes present in the *W3110* genome file, *bir*, *phnQ* (*yjdP*; b4487), *ytjA* (b4568), *ldrABC* (b4419, b4421, and b4423), and *ldrD* (b4453) have not been examined in any experimental studies, and were thus excluded from the analyses. The 76 additional open reading frames that were targeted in the Keio study, but which do not yet have designated gene names or Blattner numbers were included only in looking at the overlap of essentiality classification, and not for the ROL analyses (three of these additional open reading frames have been designated as essential by PEC). All IS elements, prophage, pseudo genes, and phantom genes were excluded.

In total, 4217 open reading frames were considered in looking at the overlaps of essentiality in *E. coli*. This set included 302 proteins classified as essential by Keio (*tnaB*, classified as essential by Keio, was excluded from the analysis, as the orf is interrupted at the 3' end in *W3110*) and 286 proteins classified as essential by PEC (all annotations are supplied in **Additional file 5**).

Of the 4217 protein coding genes for which we collected essentiality annotations, we gathered orthologue data for 4137 of them, again because 76 are novel reading frames that have only been annotated by Keio [14], and four (*dcuC*, *gatA*, *tnaB*, *rcsC* [14]) are interrupted by an IS element in *W3110*.

Functional classes. Functional classes were defined according to MultiFun annotations [15]. MultiFun divides groups of genes in several ways: by function (metabolism, information transfer, regulation, and transport), cell process (cell division, motility, adaptation to stress, and protection), cell structure (ribosome, membrane, peptioglycan, surface antigen, and flagellum) and cellular location (cytoplasm, periplasm, inner membrane, and outer membrane).

Statistical analyses. Correlations were calculated using the R statistical package v2.6.1 [16]. 95% confidence intervals for correlation coefficients were calculated by bootstrapping pairs of ROL values 100 times. Receiver operator characteristic (ROC) curves were calculated and visualized using the ROCR 1.0.2 package [17]. Significance values for the AUC values were calculated using permutation tests in which the notations of essentiality were randomized and AUC values were recalculated 1000 times.

Figure S1. Phylogenetic relationships between bacterial taxa used to infer ROL values. The phylogeny is based on a distance matrix calculated from a set of 73 highly conserved orthologues and is rooted with four archaeal species. The major bacterial taxonomic divisions are indicated. The topology that we inferred for the phylogenetic

relationships largely agrees with previous studies, although we briefly note the following contrasts: we find a weak grouping of the Actinobacteria with Deinococcales, Chloroflexi, and Cyanobacteria, in agreement with one recent study [8] but in disagreement with a second [9]. We find that the earliest branching clades are the Aquifex and Thermotoga clades, again, more closely agreeing with Pisani et al. than Ciccarelli. We find that the Spirochaetes, Chlorobi, and Chlamydiae group together [8], with Planctomycetes also within this grouping. We find the ε-proteobacteria to be the most diverged proteobacterial group; additionally, we do not find the δ-proteobacteria to be monophyletic, as the Acidobacteria appear within the clade. Finally, we note that Magnetococcus does not group strongly with any proteobacterial group, instead falling as a deeply rooted sister taxa to the α-proteobacteria, in agreement with the most recent study [10].

Figure S2. Relationship between ROL values and quantitative measurements of the effects of gene deletions (growth yield). Each point shows the ROL value calculated for the orthologue set of one *E. coli* gene (and the corresponding set of orthologues) and the measured growth yield after 22 hours in rich media. There is a small but highly significant relationship between ROL and this quantitative measure of the fitness effects of gene deletions ($r^2 = 0.0628$, $p < 0.0001$; Spearman's $\rho = 0.127$, $p < 0.0001$). The line indicates a least squares fit to the data.

Figure S3. Relationship between the relative rate of gene loss to gain and the accuracy of predicting gene essentiality. The black circles indicate the AUC values for

ROL over a range of bias parameters, from 0.5 (1:1 bias favoring gene loss over gene gain) to 0.99 (99:1 bias favoring gene loss over gene gain). The inverted white triangles indicate the AUC values for numbers of gene losses, while the black triangles show the AUC values for the numbers of gene gains. Notably, both of these measures are less accurate and less robust than ROL over a wide range of bias parameters in terms of their ability to distinguish essential and nonessential genes. The correlation between ROL and gene essentiality remains high over a wide range of parameter values, from 3:1 (0.75) to 40:1 (0.975) (black circles). Only at extreme values of the bias parameters does the accuracy of ROL in predicting gene essentiality begin to decline. The dotted line indicated the AUC value when only the fraction of taxa in which an orthologue is present is used as the metric.

1. Wall DP, Fraser HB, Hirsh AE: **Detecting putative orthologs.** *Bioinformatics* 2003, **19:**1710-1711.
2. Baba T, Ara T, Hasegawa M, Takai Y, Okumura Y, Baba M, Datsenko KA, Tomita M, Wanner BL, Mori H: **Construction of Escherichia coli K-12 in-frame, single-gene knockout mutants: the Keio collection.** *Molecular Systems Biology* 2006, **2:**2006.0008.
3. Edgar RC: **MUSCLE: a multiple sequence alignment method with reduced time and space complexity.** *Bmc Bioinformatics* 2004, **5:**1-19.
4. Talavera G, Castresana J: **Improvement of phylogenies after removing divergent and ambiguously aligned blocks from protein sequence alignments.** *Systematic Biology* 2007, **56:**564-577.
5. Felsenstein J: **PHYLIP - Phylogeny Inference Package (version 3.6).** *Cladistics* 2005, **5:**164-166.
6. Desper R, Gascuel O: **Fast and accurate phylogeny reconstruction algorithms based on the minimum-evolution principle.** *Journal of Computational Biology* 2002, **9:**687-705.
7. Rambaut A: **FigTree.** 2006.
8. Pisani D, Cotton JA, McInerney JO: **Supertrees disentangle the chimerical origin of eukaryotic Genomes.** *Molecular Biology and Evolution* 2007, **24:**1752-1760.
9. Ciccarelli FD, Doerks T, von Mering C, Creevey CJ, Snel B, Bork P: **Toward automatic reconstruction of a highly resolved tree of life.** *Science* 2006, **311:**1283-1287.
10. Esser C, Martin W, Dagan T: **The origin of mitochondria in light of a fluid prokaryotic chromosome model.** *Biology Letters* 2007, **3:**180-184.
11. Bollback JP: **SIMMAP: Stochastic character mapping of discrete traits on phylogenies.** *Bmc Bioinformatics* 2006, **7:**88.

12. Huelsenbeck JP, Nielsen R, Bollback JP: **Stochastic mapping of morphological characters.** *Systematic Biology* 2003, **52:**131-158.
13. Kato JI, Hashimoto M: **Construction of consecutive deletions of the Escherichia coli chromosome.** *Molecular Systems Biology* 2007, **3:**132.
14. Hayashi K, Morooka N, Yamamoto Y, Fujita K, Isono K, Choi S, Ohtsubo E, Baba T, Wanner BL, Mori H, Horiuchi T: **Highly accurate genome sequences of Escherichia coli K-12 strains MG1655 and W3110.** *Molecular Systems Biology* 2006, **2:**2006.0007.
15. Serres MH, Riley M: **MultiFun, a multifunctional classification scheme for Escherichia coli K-12 gene products.** *Microb Comp Genomics* 2000, **5:**205-222.
16. R Development Core Team: **R: A Language and Environment for Statistical Computing.** 2007.
17. Sing T, Sander O, Beerenwinkel N, Lengauer T: **ROCR: visualizing classifier performance in R.** *Bioinformatics* 2005, **21:**3940-3941.

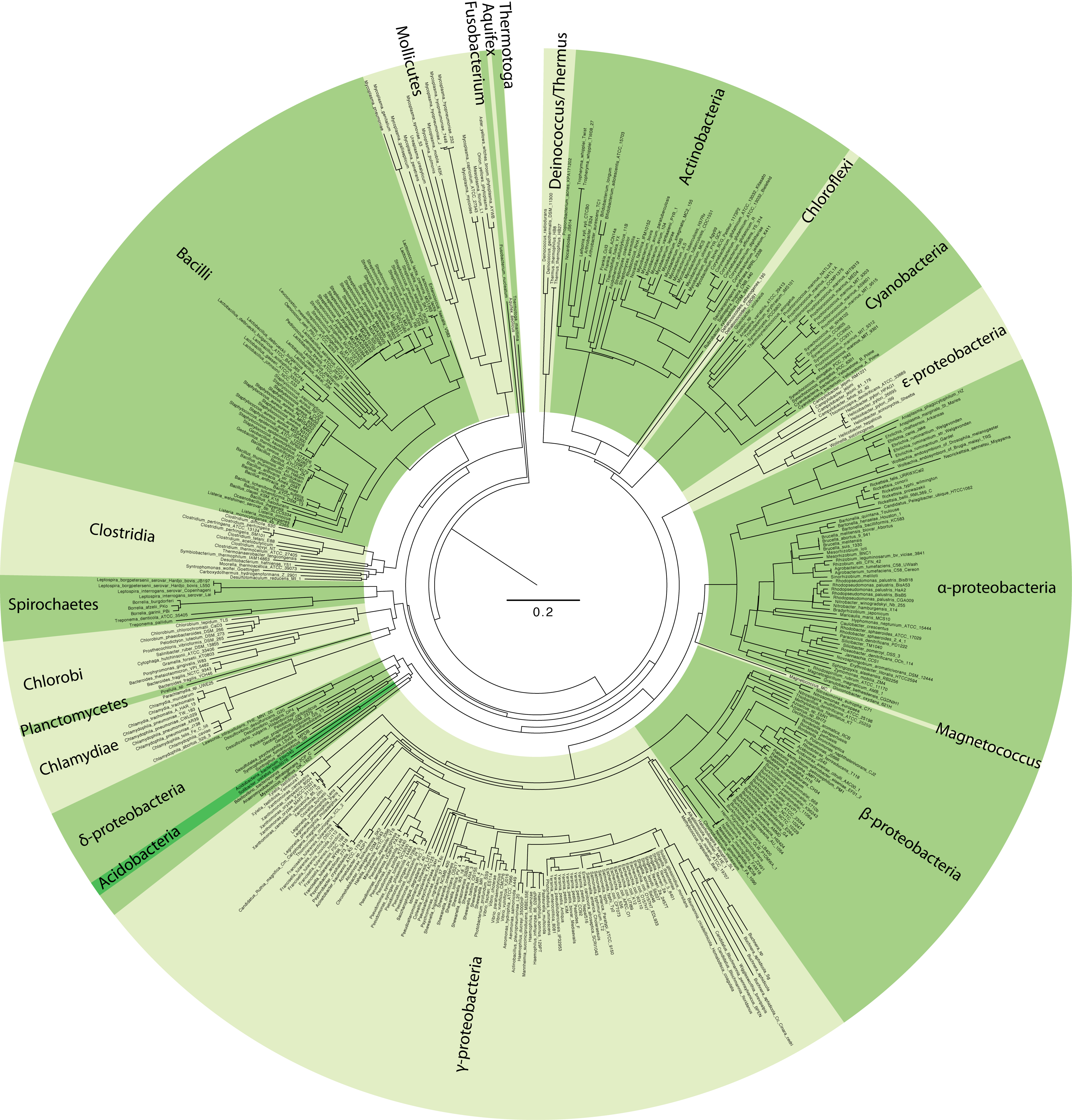

Thermotoga
Aquifex
Fusobacterium
Mollicutes
Deinococcus/Thermus
Actinobacteria
Chloroflexi
Cyanobacteria
ε-proteobacteria
α-proteobacteria
Magnetococcus
β-proteobacteria
γ-proteobacteria
Acidobacteria
δ-proteobacteria
Chlamydiae
Planctomycetes
Chlorobi
Spirochaetes
Clostridia
Bacilli
0.2

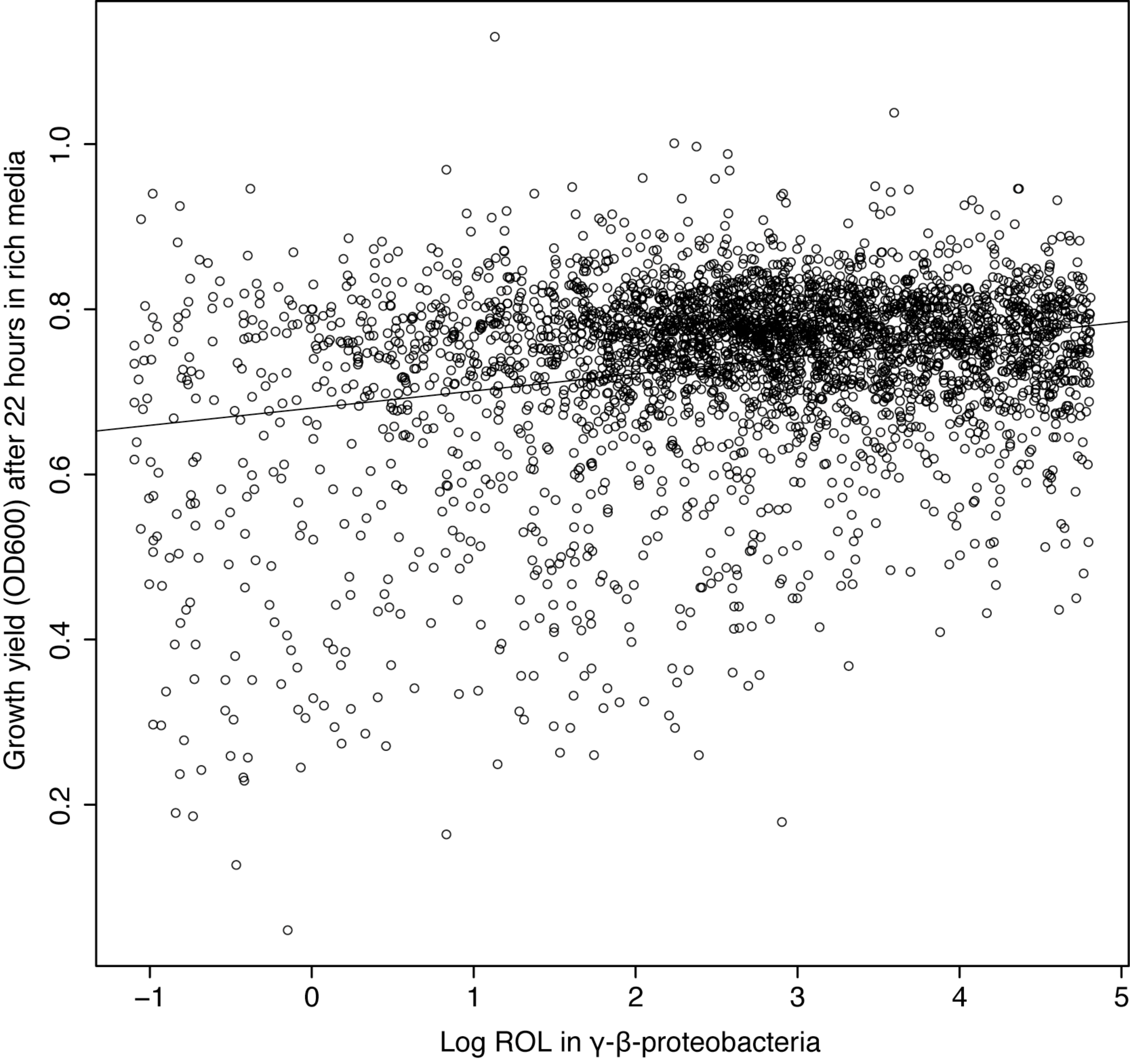
Growth yield (OD600) after 22 hours in rich media
Log ROL in γ-β-proteobacteria
0.2
0.4
0.6
0.8
1.0
−1
0
1
2
3
4
5

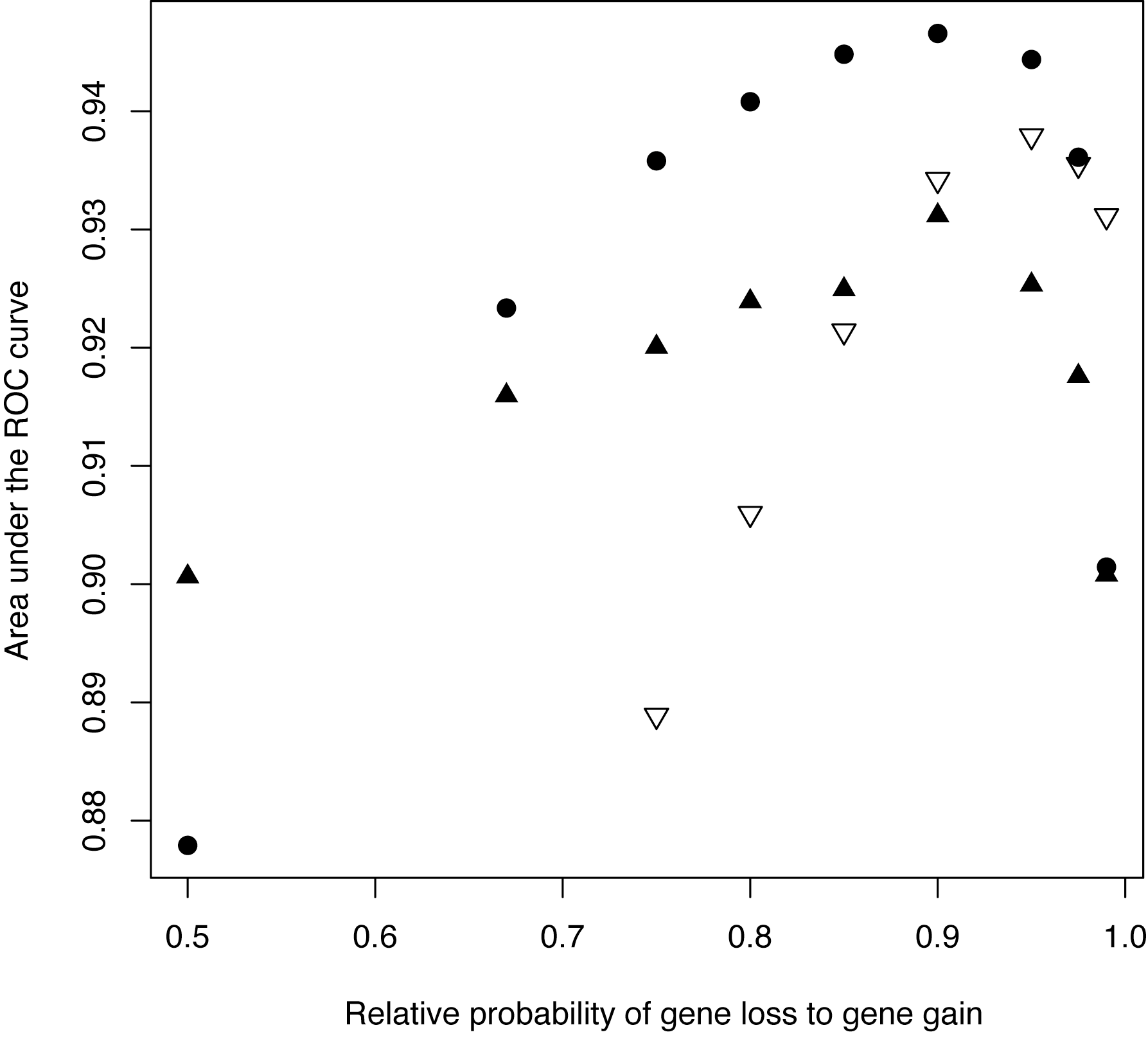
Area under the ROC curve
0.88
0.89
0.90
0.91
0.92
0.93
0.94
0.5
0.6
0.7
0.8
0.9
1.0
Relative probability of gene loss to gene gain
